# The Good, the Bad and the Hijab: A Study of Implicit Associations Made by Practicing Muslims in Their Native Muslim Country

**DOI:** 10.1177/00332941221103532

**Published:** 2022-05-19

**Authors:** Mercedes Sheen, Hajar Aman Key Yekani, Timothy R. Jordan

**Affiliations:** Department of Psychology, 150991 Heriot-Watt University, Dubai, UAE; Department of Psychology, 448243 Ibn Haldun University, Istanbul, Turkey

**Keywords:** Person perception, culture, implicit association test, physical attractiveness stereotype, hijab

## Abstract

Recent research indicates that wearing the hijab reduces the attractiveness of female faces perceived by practicing Muslim men and women in their native Muslim country (the United Arab Emirates). The purpose of the current research was to develop this finding to investigate whether other aspects of person perception are also affected when women wear the hijab in this Muslim country. Of particular relevance is that changes in physical attractiveness often affect the personal qualities assigned to individuals. Accordingly, we sought to determine whether such effects occur when the physical attractiveness of women is altered by wearing the hijab. To do this, we used an Implicit Association Test (IAT) to investigate how native Muslim participants in the UAE associated pleasant and unpleasant connotations with images of women either wearing the hijab or with their heads uncovered. As in previous research with native Muslim participants, female faces were again perceived as significantly less attractive when the hijab was worn. However, the accompanying IAT findings showed that these less attractive hijab-wearing images were associated more with pleasant connotations than were the matched uncovered images. These findings provide fresh insight into the effects of the hijab on perceptions of Muslim women in a Muslim country and provide support for the view that cultural clothing can influence person perception beyond physical attractiveness alone.

The hijab, meaning “barrier” in Arabic, is a religious garment worn for many reasons by Muslim women throughout the world, including as a display of their modesty and religious identity. But although countless Muslim women wear the hijab, relatively little is known about the effects this garment actually exerts on how these women are perceived by others.

In recent years, several studies (Jordan et al., 2020; Mahmud & Swami, 2010; Pasha-Zaidi, 2015; Sheen et al., 2018; Swami, 2012) have investigated how the hijab affects perceptions of facial attractiveness. The earlier of these studies (Mahmud & Swami, 2010; Pasha-Zaidi, 2015; Swami, 2012) were conducted in predominantly non-Muslim countries (UK, USA) and show some indication that the hijab lowers the perceived facial attractiveness of the wearer. But these results are mixed and may well have been affected by anti-Islamic feelings in the countries concerned (for discussions, see Jordan et al., 2020; Mahmud & Swami, 2010; Sheen et al., 2018; Swami, 2012). Research conducted more recently (Jordan et al., 2020; Sheen et al., 2018) investigated the effects that wearing the hijab produced on the facial attractiveness of women perceived by practicing Muslims resident in their native Muslim country (the United Arab Emirates; hereafter UAE), where the hijab is a normal aspect of everyday life and where anti-Islamic attitudes should not affect participants’ judgments. In both these studies, the attractiveness of female faces was rated in two key conditions: heads were fully covered by the hijab except for the face or heads were completely uncovered. The findings revealed that male and female participants rated facial attractiveness in these conditions lower when the hijab was worn compared to when heads were uncovered, suggesting that the hijab does indeed reduce the physical attractiveness of the wearer in Muslim societies.

However, these effects of the hijab on perceptions of female facial attractiveness may reflect just part of the influence of this garment in Muslim societies. Of particular relevance is that a major finding in psychological research is that people who are perceived as physically more attractive are often assumed to also possess more socially desirable qualities than their less attractive counterparts (e.g., Little et al., 2011). Dion et al. (1972) were among the first researchers to investigate this effect (often called the physical attractiveness stereotype, hereafter PAS) under laboratory conditions and found that people are more likely to assign positive qualities to more physically attractive people and negative qualities to less physically attractive people. Later studies by Eagly et al. (1991) and Feingold (1992) established the PAS further as a robust form of cognitive bias and showed that facial attractiveness influences many aspects of person perception, including the increased attribution of positive qualities like sincerity, honesty, competence, and intelligence to more attractive individuals (e.g., Little et al., 2011). Indeed, physical attractiveness is among the first observable cues used to make judgments about others (e.g., see the primacy effect; Anderson & Barrios, 1961) and evidence suggests that once an initial positive impression about a person is formed, subsequent information that is congruent with that expectancy (this person is good) is processed more fluently than subsequent information that is incongruent (this person is bad; Bruin, 2005). Accordingly, in view of the evidence that wearing the hijab reduces the physical facial attractiveness of women perceived by practicing Muslims resident in their native Muslim country, wearing the hijab may also affect the personal qualities attributed to wearers of this religious garment.

Although there is much evidence to indicate that cues from clothing can influence how women are perceived (for a review, see Johnson et al., 2014), the effect of wearing the hijab on the attribution of personal qualities in native Muslim society provides a special cultural condition that has yet to be explored. For example, although some evidence exists in the literature that the hijab may negatively influence interpersonal judgments (Swami, 2012), this evidence was obtained with participants in a non-Muslim country. Swami asked both Muslim and non-Muslim men living in the UK to rate head images of women for attractiveness and a number of personal qualities when the women were wearing the hijab and when they were wearing no hijab at all (uncovered). The results indicated that, compared to uncovered images, images of women wearing the hijab were rated by both Muslim and non-Muslim males as more religious but less attractive, less approachable, less competent, less sociable, and less popular. These findings provide a useful indication that wearing the hijab can negatively affect the attribution of personal qualities to the wearer. But as this research was conducted in the UK, where wearing the hijab is rare in what is a largely non-Muslim national population, it remains unclear whether the hijab in this study inspired anti-Islamic feelings which then produced these lower ratings, even for Muslim participants (for further discussion of this issue, see Swami, 2012; see also the Turban Effect, Unkelbach et al., 2008). A further complication with this earlier research is that different photographs were used for each of the two types of display (hijab and uncovered). Unfortunately, it is well established that even slight changes in facial appearance can alter substantially the processing of a face, including its perceived attractiveness (e.g., Perrett et al., 1994; Little et al., 2011), and so it is difficult to be certain about the effect that the hijab may have had on the judgments made in this earlier study (for further discussion of the importance of facial matching in experiments of this kind, see Jordan et al., 2020; Sheen et al., 2018). More research is needed to help develop a clearer understanding of the nature and extent of the effect that the hijab produces on perceptions of the personal qualities of wearers of this garment, especially in native Muslim society.

A crucial step forward in this respect is to investigate the effect of wearing the hijab on the attribution of personal qualities made by practicing Muslim citizens in their native Muslim country. In particular, given the immense cultural importance of the hijab and its demonstrated effectiveness at reducing facial attractiveness in Muslim society, it remains to be seen how wearing the hijab affects the personal qualities attributed to wearers of this garment in a Muslim country. This is particularly relevant since the PAS suggests that changes in physical attractiveness affect the personal qualities attributed to individuals and, therefore, that wearing the hijab may inspire the attribution of negative qualities to the wearer. But most studies that have investigated the PAS have been conducted in non-Muslim individualistic cultures (Shaffer et al., 2000; Wheeler & Kim, 1997) where identity is based primarily on personal, individuating attributes (Hofstede & Bond, 1984; Hofstede et al., 2010). Moreover, Dion et al. (1990) proposed that the PAS should be less prevalent in collectivistic, group-oriented cultures (like the UAE; Hofstede et al., 2010; see also Jordan et al., 2020; Jordan et al., 2021), because identity in these societies is based less on characterological inferences than perception of group affiliation. In their study, Dion et al. asked members of the Chinese community living in Canada to rate the personality traits and expected life outcomes of images of Chinese people with various levels of physical attractiveness. Their findings indicated that participants who reported high involvement with their Chinese community, and thus greater identification with their culture, were less likely to make interpersonal judgments based on physical attractiveness of the stimulus persons than participants who reported low involvement with their Chinese community. Dion et al. (1990) argued that the sociocultural perspective mitigates against the PAS so that interpersonal judgements in collectivist societies are more likely to be based on perception of salient cultural values rather than physical attractiveness. More recently, however, Chen et al. (1997) proposed a revised socio-cultural perspective and suggested that people from all cultures are vulnerable to the PAS. In a study of Taiwanese participants, living in the collectivist culture of Taiwan, they found participants relied on facial attractiveness cues when making characterological inferences about attractive and unattractive Taiwanese faces, especially when those judgements activated communal cultural values. However, in a later cross-cultural study of American and Taiwanese undergraduates, Shaffer et al. (2000) found that although PAS was shown readily in both cultures, Taiwanese participants were even *more* inclined than American participants to make positive evaluations of own-race attractive faces, irrespective of whether those evaluations activated collectivistic or individualistic value orientations. In view of these different perspectives, the Muslim collectivistic culture of the UAE provides a valuable opportunity for obtaining fresh insight into the influence of the hijab on perception of the individual.

Against this background, the purpose of the current research was to investigate the effects of the hijab on perceptions of the personal qualities of the wearer made by practicing Muslims resident in their native Muslim country. If the lowered facial attractiveness produced by the hijab in Muslim society (and observed previously; Jordan et al., 2020; Sheen et al., 2018) also affects the personal qualities associated with the wearer in ways that are consistent with the PAS, the personal qualities associated with individuals wearing the hijab should be more negative compared to when no hijab is worn, which is what we would expect with non-Muslim participants outside of the UAE.

However, the hijab is a religious garment of immense cultural importance to Muslims, especially those native to and living in the UAE (Koornneef et al., 2017). Moreover, because the hijab represents “*Emirati-ness*” (Sulayman, 2005; Trainer, 2017), and adherence to a shared faith and common social identity (Bristol-Rhys, 2010; Koornneef et al.), observing a woman wearing the hijab is likely to arouse feelings of in-group membership and belongingness (for a discussion, see Grey & Thomas, 2018). Accordingly, since the sociocultural perspective suggests that interpersonal judgments in collectivist societies are based on perception of group affiliation, we hypothesized that although the hijab would lower perceived facial attractiveness, the hijab would produce greater associations with pleasant connotations compared to when the wearer is uncovered. In this case, the indication would be that the effect of the hijab on perceptions of hijab wearing Muslim women made by Muslims in their native Muslim country does indeed extend beyond perception of facial attractiveness and affects how personal connotations are associated with the wearer in Muslim society, in ways that overcome influences of the PAS.

Following previous work on associations between physical attractiveness and attributions of personal qualities, we used a version of the Implicit Association Test (IAT; Greenwald et al., 1998) which was designed to assess implicit associations of pleasant (e.g., “happy”) and unpleasant (e.g., “nasty”) connotations with faces of hijab-wearing and uncovered females. Participants were shown two types of visual stimulus, either adult female faces (shown wearing the hijab or uncovered) or individual words representing particular connotations (pleasant or unpleasant). Each type of stimulus was shown individually, and, on each trial, participants were required to categorize each face or word as quickly as possible using two response options that paired faces and words in different ways. In one response condition, response options were “uncovered or pleasant” and “covered or unpleasant” while in the other response condition, response options were “covered or pleasant” and “uncovered or unpleasant”. The logic of this approach is that response combinations of faces and connotations that are more associated in memory (e.g., uncovered face and pleasant connotations, covered face and unpleasant connotations) should be easier to process than the reverse combinations (covered face and pleasant connotations, uncovered face and unpleasant connotations), and this processing difference should be revealed by faster reaction times for the response combinations that are regarded as more closely associated by participants (see Greenwald et al. for further discussion).

## Method

### Ethics Statement

This study was approved by the Research Ethics Committee of a government university in the UAE, and informed, written consent was obtained from all participants.

### Participants

A total of 96 Arabic UAE nationals (48 females), aged 18–28, took part in this study and were recruited via the use of flyers posted in the UAE. All participants reported being Arabic, practicing Muslims born and resident in the UAE, and this information was verified using official documentation and one-to-one interviews. As expected in this society, responses to the DUREL index of religious involvement (Koenig & Büssing, 2010) indicated that participants were highly religious, with a mean of 4.52 out of a maximum of 5 (mode = 4.78; range = 3.3–5).

It is already established that, at a nation level, the UAE scores high in collectivism (Hofstede et al., 2010). However, to ensure that this cultural orientation existed for the individuals taking part in this experiment, all participants completed the 16-item Individualism/Collectivism scale (Triandis & Gelfand, 1998) with responses based on a 1 (*strongly disagree*) to 7 (*strongly agree*) range. The results indicated that, for each participant, scores for collectivism were much higher than scores for individualism, and these differences were confirmed through Bonferroni-corrected t-tests, *p* < .001, for males (*M* = 6.36, *SD* = .42 vs. *M* = 3.20, *SD* = .53) and females (*M* = 6.22, *SD* = .33 vs. *M* = 2.55, *SD* = 1.09). These results are consistent with nation-level scores for collectivism reported previously (see Hofstede, et al., 2010) and support previous findings concerning the pervasiveness of collectivism in the UAE (Hofstede et al., 2010; Zeffane, 2020). Finally, Bailey-Lovie assessments (Bailey & Lovie, 1980; see; Jordan et al., 2011) determined that participants had normal (or corrected-to-normal) visual ability.

Forty-eight (24 females) of the 96 participants were selected randomly to take part in the IAT. However, we also wished to check that the covered and uncovered faces used in the IAT showed the same pattern of facial attractiveness indicated by previous studies (Jordan et al., 2020; Sheen et al., 2018; see also; Mahmud & Swami, 2010; Pasha-Zaidi, 2015; Swami, 2012). Accordingly, the remaining 48 participants (24 females) from the same population as those taking part in the IAT rated the facial attractiveness of the stimuli used in the IAT in a separate Attractiveness Rating Task (ART). This procedural discretion was important because it helped conceal from participants the potential link between assignment of personal qualities and facial attractiveness, and so helped avoid cross-contamination between these two sections of the experiment (see Swami & Hull, 2009, for compelling endorsement of this procedure in attractiveness research). A priori estimates of the required sample size for the IAT and the ART were obtained using G*Power (Faul et al., 2007) for a statistical power of .95, an alpha level of .05, and a medium effect size of *d* = .50. The power analysis indicated that a sufficient sample for each section (IAT, ART) would be 34 participants, indicating that our samples of 48 were appropriately powered.

### Stimuli

**Figure 1.**
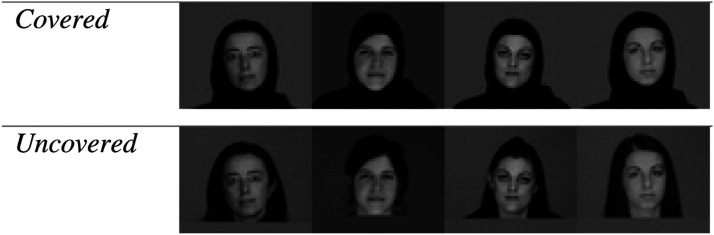
Examples of the facial stimuli used.

The IAT involved presenting connotations (pleasant or unpleasant words) and female facial stimuli (covered or uncovered) individually on a screen. Connotations were provided by 8 pleasant words (joy, love, peace, wonderful, pleasure, glorious, laughter, happy) and 8 unpleasant words (agony, terrible, horrible, nasty, evil, awful, failure, hurt) that are used commonly in the IAT (e.g., Bellezza et al., 1986). All words were presented in 14-point Courier typeface. Facial stimuli were provided by frontal views of the heads of 8 Muslim women (see Figure 1). Each woman was photographed both wearing her hijab and uncovered. The face of each woman was presented in two display conditions: in the *covered* condition, each head was covered completely by the hijab except for the face, whereas, in the *uncovered* condition, no hijab was worn (Figure 1). For each woman, it was important to use exactly the same facial image in both display conditions as even slight changes in pose and expression may affect perceptions of facial attractiveness (see Sheen et al., 2018, for discussion). Consequently, to ensure precise facial matching, the covered stimulus for each woman was formed by digitally superimposing the hijab onto the uncovered stimulus. In this way, two display conditions were produced for each woman whereby the face was unchanged except for the effect of the presence or absence of the hijab. All facial stimuli were presented full-size and in color and all were presented in both the IAT and in the ART. At the end of the experiment, participants were asked whether the images looked natural and all participants reported that they were unaware that the hijab had been superimposed and confirmed that the images all looked natural.

### Apparatus

Both parts of this study (IAT, ART) were conducted on an Apple Macintosh 3.7 GHz computer using SR Experiment Builder software, and all stimuli were presented on an Apple Macintosh 27-inch High Definition display screen. Stimulus presentations were synchronized with the refresh cycle of the monitor and participants made responses via an SR response box interfaced with the computer which provided millisecond response-timing accuracy.

### Design and Procedure

All 48 participants who took part in the IAT were shown all stimuli (facial images, connotations) individually, presented in a different random order for each participant. Twelve practice stimuli were shown at the start of each session to familiarize participants with the procedure. Each stimulus in the IAT was presented in the middle of the screen and participants were required to categorize the face or word presented on each trial using two keys interfaced with the computer. Responses took place in two association conditions. In one association condition, participants were required to use one key to respond “uncovered or pleasant” and the other key to respond “covered or unpleasant”. In the other association condition, these pairings were switched so that participants were required to use one key to respond “covered or pleasant” and the other key to respond “uncovered or unpleasant”. In this way, pairings of faces and connotations that are more associated in memory should be easier to process and be revealed by faster reaction times than the less associated combinations. The response options corresponding to each response association condition (uncovered or pleasant/covered or unpleasant OR covered or pleasant/uncovered or unpleasant) were indicated by labels in the upper left and upper right corners of the screen, corresponding to the left and right positions of two response keys placed in front of each participant. For each response association condition, response options were counterbalanced across the two response keys. Following previous IAT procedures, all participants took part in both response association conditions, and the order of these conditions was counterbalanced across participants. Participants took part individually in a sound-attenuated room and sat at a distance of 60 cm from the display screen. Each stimulus was presented one at a time in the centre of the screen and participants were required to categorize the face or word presented on each trial. Participants were informed that their reaction time was of utmost importance and they should respond as quickly as possible whilst trying to avoid making mistakes.

The 48 participants who took part in the ART were shown the same facial stimuli used in the IAT under the same viewing conditions and rated the facial attractiveness of each stimulus as in other research (see Jordan et al., 2020; Sheen et al., 2018). In line with these previous studies, each participant was shown each facial stimulus displayed one at a time, in a random order, with a 7-point scale that was used to rate the attractiveness of each face (1 = *very unattractive* to 7 = *very attractive*). Participants were instructed to rate each image carefully and responded on each trial by pressing one of 7 keys interfaced with the computer, corresponding to the 7-point scale. Each image remained on the screen until a response had been made, after which the next image was presented.

## Results

**Figure 2.**
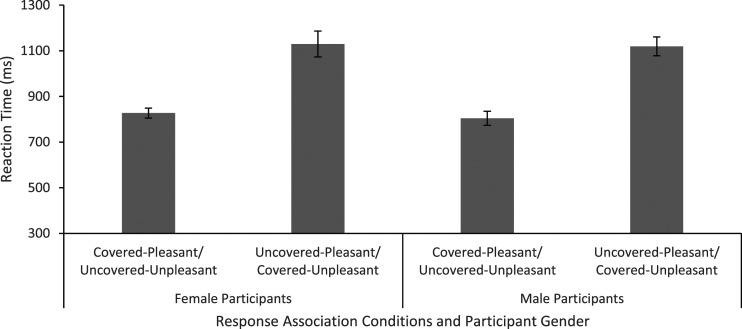
Mean reaction times (with Standard Error bars) for response association conditions and participant gender in the IAT.

For scoring performance on the IAT, the strength of association between faces and connotations was determined by comparing reaction times for different types of face-connotation combinations, such that a combination that produced faster reaction times indicated a stronger face-connotation association. For example, if participants associated covered faces with pleasant connotations and uncovered faces with unpleasant connotations, covered or pleasant/uncovered or unpleasant response options should be processed more efficiently and produce faster reaction times than covered or unpleasant/uncovered or pleasant response options (see Method). Mean reaction times for each participant gender (male, female) and response association condition (covered - pleasant/uncovered - unpleasant; uncovered - pleasant/covered - unpleasant) are shown in Figure 2. A mixed design ANOVA, with factors participant gender and response association condition, showed no main effect of participant gender, *F*(1,46) = .12, *p* = .73, $\eta_{p}^{2}$ = .003, but did show a main effect of response association condition, *F*(1,46) = 105.03, *p* < .0001, $\eta_{p}^{2}$ = .70, reflecting faster reaction times for covered - pleasant/uncovered - unpleasant responses (*M* = 816 ms) than for uncovered – pleasant/covered - unpleasant responses (*M* = 1125 ms). No interaction between participant gender and response association condition was found, *F*(1,46) = .05, *p* =.83, $\eta_{p}^{2}$ = .001.

Some exploratory analyses were conducted to see whether IAT responses differed based on the attractiveness of the faces. For this purpose, only the response times for participants’ exposure to faces were included in the analyses. In addition, the face stimuli were divided into low and high attractiveness based on their ART ratings. A repeated measures ANOVA, with factors attractiveness (low, high) and response association condition, showed no main effect of attractiveness, *F*(1,58) = .86, *p* = .36, $\eta_{p}^{2}$ = .015, but did show a main effect of response association condition, *F*(1,58) = 12.89, *p* < .01, $\eta_{p}^{2}$ = .18, reflecting faster reaction times for covered or pleasant/uncovered or unpleasant responses (*M* = 751 *ms*) than for covered or unpleasant/uncovered or pleasant responses (*M* = 1052 *ms*). No interaction between participant gender and response association was found, *F*(1,58) = 1.20, *p* =.28, $\eta_{p}^{2}$ = .02.

Implicit Association Test responses were analysed further using Greenwald, Nosek and Banaji’s (2003) scoring algorithm. Instead of comparing within-person differences in raw latencies, these differences were standardized at the participant level by dividing differences between the mean response latencies of the two combined tasks by the *SD* of all latencies in these tasks. In this case, the strength of the IAT effect corresponds to conventional criteria used to define small (.20), moderate (.50), and large (.80) effect sizes of Cohen’s *d* measure. The overall mean observed *d*-score for reaction time was 1.25 (*SD*=.84), and this was significantly different from 0, *t* (47) = 10.35, *p* < .001, 95% *CI* [1.01, 1.49].

To determine that the relative attractiveness of the facial stimuli used in the IAT was the same as in previous research, the attractiveness ratings from the ART were submitted to a mixed design ANOVA, with factors participant gender (male, female) and display condition (covered, uncovered). No main effect of participant gender was found, *F*(1, 46) = 3.24, *p* = .08, $\eta_{p}^{2}$ = .07, but the main effect of display condition was significant, *F*(1, 46) = 8.26, *p*<.01, $\eta_{p}^{2}$ = .15, reflecting higher attractiveness ratings for uncovered faces (*M* = 3.79) than for covered faces (*M* = 3.28), as in previous research (see Jordan et al., 2020; Sheen et al., 2018). No interaction between participant gender and display condition was found, *F*(1, 46) = .10, *p* = .75, $\eta_{p}^{2}$ = .002.

The purpose of this research was to investigate the responses made by practicing Muslims native to and living in the UAE, and all participants showed the high levels of religiosity and collectivism (see *Participants* section) that are typical in this society (e.g., Hofstede & Bond, 1984; Hofstede et al., 2010). This consistency underscores the relevance of the findings of this research, but these characteristically high levels of religiosity and collectivism made it unlikely that there would be sufficient variation to show a fine-scale influence of religiosity and collectivism on performance in the IAT and ART. Nevertheless, for completeness, we investigated these possibilities. Turning first to religiosity, a Shapiro-Wilk test of distribution normality for DUREL religiosity scores was significant for males, *S-W* = .77, *df* = 24, *p* < .001, and females, *S-W*=.86, *df*=24, *p <* .005, reflecting a skew towards high religiosity scores. Accordingly, a Spearman correlation coefficient was used to examine the association between DUREL scores and IAT and ART responses. For IAT responses, no significant correlation with DUREL scores was found, for males or females, for either response association condition (covered-pleasant/uncovered-unpleasant; uncovered-pleasant/covered-unpleasant; for both conditions, *r*s < .23, *p*s > .28). Similarly, for ART ratings, no significant correlation with DUREL scores was found, for males or females, for either display condition (covered, uncovered; for both conditions, *r*s < .27, *p*s > .20).

For collectivism scores, a Shapiro-Wilk test of distribution normality was significant for males, *S-W*=.89, *df* = 48, *p* < .001, and females, *S-W* = .78, *df* = 48, *p* < .001, reflecting a skew towards high collectivism scores. Consequently, Spearman correlation coefficients were used to examine the association between collectivism scores and IAT and ART responses. For IAT responses, no significant correlation with collectivism scores was found, for males or females, for either response association condition (for both conditions, *r*s < .26, *p*s > .22). For ART ratings, no significant correlation with collectivism scores was found, for males or females, for either display condition (for both conditions, *r*s < .25, *p*s > .26).

## Discussion

The present study replicates previous findings that Muslim males and females native to and living in a Muslim country perceive faces of women wearing the hijab as less attractive than the same faces shown when the head is uncovered (Jordan et al., 2020; Sheen et al., 2018). But the study now extends these previous findings to show that, using exactly the same facial stimuli, participants are more likely to associate pleasant connotations with facial images shown when wearing the hijab and unpleasant connotations with the same facial images shown when the head is uncovered. These findings support the hypothesis that the effect of wearing the hijab extends beyond perceptions of facial attractiveness alone, and provide new information concerning the ways in which wearing the hijab influences how Muslim females are perceived by practicing Muslims in their native Muslim country.

While the hijab is clearly effective at reducing how attractive a woman’s face is regarded (see also the findings of Jordan et al., 2020; Sheen et al., 2018), the nature of the IAT findings suggests that this negative effect is not reflected straightforwardly in the personal qualities associated with the wearer. In particular, if facial attractiveness is a major determinant of how people are perceived, wearing the hijab may be expected to elicit a greater association with unpleasant connotations relative to when no hijab is worn. But essentially the opposite effect was observed, showing instead that the same hijab-wearing images that produced lower levels of facial attractiveness also produced a greater association with pleasant connotations.

There are two complementary explanations for this effect. The first is that perceived lower facial attractiveness when wearing the hijab is regarded positively by practicing Muslims as this effect is an intended culturally-driven consequence of wearing this garment, and so there is no cognitive inconsistency between the lower facial attractiveness observed for hijab-wearing faces and the association of pleasant connotations with these images. The hijab is a symbol of piety and devotion to faith and is often regarded as a means for women to reduce their attractiveness to males outside their immediate family, in line with the writings of the *Qur’an* (e.g., verse 24:31; see Noor, 2009). Practicing Muslims are very aware of this purpose (as were the highly religious participants in this study) and so the lowered attractiveness ratings observed when the hijab was worn may reflect an important form of self-fulfilling prophecy (Merton, 1948) in this process. Indeed, because the hijab symbolizes adherence to a shared faith and common social identity (Bristol-Rhys, 2010; Koornneef et al., 2017), wearing the hijab may also inspire feelings of in-group membership, which may also have contributed to the association of pleasant connotations with hijab-wearing faces that was observed. So, in this sense, the lowered facial attractiveness of faces observed when wearing the hijab is cognitively consistent with other cultural considerations and both sources of cultural influence generate positive perceptions of the personal qualities of the wearer.

The second explanation is that perceived lower facial attractiveness when wearing the hijab is regarded negatively by practicing Muslims and so there is a cognitive inconsistency between the lower facial attractiveness produced by hijab-wearing faces and the association of pleasant connotations with these images. For example, it has been demonstrated consistently that the hijab reduces facial attractiveness (e.g., Jordan et al., 2020; Sheen et al., 2018) and this negative effect may reflect the disruption of normal processes of perceiving human facial attractiveness when external features of the face (e.g., the hair and ears) are occluded (e.g., Toseeb et al., 2014; Toseeb et al., 2012; see also discussions by; Sheen et al., 2018). However, despite producing lower attractiveness ratings, these negative perceptual effects may also be offset by the perception of in-group membership described above, as the presence of the hijab nevertheless still symbolizes positive associations, leading to the association of pleasant connotations with faces wearing the hijab. In this sense, the lowered facial attractiveness of faces that is actually perceived when wearing the hijab is cognitively inconsistent with powerful cultural considerations, but it is these powerful cultural considerations that dominate perception of the personal qualities of the wearer.

The PAS suggests that people who are perceived as physically more attractive are assumed to also possess more desirable attributes than their less attractive counterparts (e.g., Dion, et al., 1972; Little et al., 2011). The findings presented in the current study question this view and support the notion that culture plays a moderating role in how the PAS operates in different cultural settings. Moreover, the sociocultural perspective (Dion, et al., 1990) argues that people from group-oriented, collectivistic cultures (see Hofstede & Bond, 1984; Hofstede, et al., 2010) are more likely to make interpersonal judgments based on perceived group membership of a person rather than individuating cues (such as facial attractiveness). This is consistent with the current research where participants showed high levels of collectivism, reflecting the UAE’s high national level on the collectivism scale (see Hofstede, et al., 2010). So, while the PAS is likely to exist in collectivistic societies (Wheeler & Kim, 1997), the stereotype may not operate wholly on the basis of physical attractiveness *per se*, but be influenced by perception that a person possesses traits that are valued highly in that particular culture (Dion, et al., 1990); what might more appropriately be called the *Cultural Attractiveness Stereotype.* Indeed, it may be that the faster reaction times observed when covered faces were paired with positive words also reflect a cultural form of perceptual fluency where the ease with which people process common, prototypical stimuli (e.g., Reber et al., 2004) is associated with positive social evaluations (e.g., Lick & Johnson, 2015). However, Emirati females make up just 3% of the total population of the UAE (Puri-Mirza, 2020), so it is unclear whether the social environment in which our participants normally circulated was sufficient to create this form of perceptual fluency. Further research on this specific topic is now needed to find empirical evidence for the notion that perceptual fluency may elicit positive affect with different levels of head and face coverings in Muslim society.

If the presence of the hijab signifies belongingness, does *not* wearing the hijab signify not belonging? The sociocultural perspective may be useful in explaining why the presence of the hijab causes participants to associate pleasant connotations with covered faces, but it doesn’t fully explain why participants associate unpleasant connotations with uncovered faces (for similar logic, see Griffin & Langlois, 2006). Preserving positive social identity is of particular importance in the UAE (Grey & Thomas, 2018) and a core concept of social identity theory (Tajfel & Turner, 1986), so it seems likely that the presence of hijab-generated perceptions of in-group identity cause faces of women not wearing the hijab to be associated more with unpleasant connotations (e.g. outgroup derogation; for a discussion see Cuddy et al., 2009) than the same faces wearing the hijab. Indeed, the “black sheep effect” (Marques & Paez, 1994) predicts that non-conforming members of an in-group (for the present purposes, those not wearing the hijab) are more likely to be appraised negatively than non-conforming members of an out-group, and this is likely to be a contributory factor to the effects we observed.

The results reported here differ greatly from those of Swami (2012) who found both Muslim and non-Muslim men in the UK rated images of women wearing the hijab less favourably across a range of interpersonal attributes than images of the same women uncovered. Although these findings provide a useful addition to the literature, the differences between Swami’s results and those reported here highlight the potential problems of conducting research on the effect of the hijab on person perception in non-Muslim societies. In the UK negative perceptions of Islamic symbols, such as the hijab, have been shown to arouse anti-Islamic feelings in a post-911 and 7/7 climate (Zempi, 2020). Indeed, Swami suggested that internalised Islamophobia may well have affected the results observed in his study. The study reported offers a more reliable measure of the way in which the hijab affects person perception in the absence of anti-Islamic influences. Indeed, although research on the effect of the hijab is most likely to be revealed by Muslims living in their native Muslim country, where participants’ judgments should not be influenced by anti-Islamic feelings, future research could test these assertions by using the same methodology in both a Muslim and non-Muslim context.

Finally, it should be noted that the use of IAT in psychological research is not without controversy. The IAT has been cited close to five thousand times in the literature yet there remains lively debate as to its use. Some argue that its test-retest reliability (Gawronski et al., 2017) and construct validity (Schimmack, 2021) cannot be established. Others argue that, despite the demand for further validation, the IAT offers the best measure of implicit attitudes currently available (Vianello & Bar-Anan, 2021). Indeed, the IAT is highly reliable at the aggregate level and consistently produces very similar results across groups of people from the same population (Jost, 2019; for further discussion see; Greenwald & Lai, 2020). Accordingly, the use of the IAT is entirely appropriate for the research reported here.

In sum, the goal of this study was to establish whether wearing the hijab affects aspects of person perception by practicing Muslims living in their native Muslim country that are beyond facial attractiveness. In line with previous work (e.g., Jordan et al., 2020; Sheen et al., 2018), faces were again rated as less attractive when the hijab was worn. But these findings were extended to show that, despite these differences in attractiveness, wearing the hijab is associated more with pleasant connotations and not wearing the hijab is associated more with unpleasant connotations. This pattern suggests that Muslim citizens in their native Muslim country, for whom the hijab is a normal aspect of everyday life, do not make personal attributions based on physical attractiveness alone, but are influenced greatly by cultural factors like perceptions of in-group social identity, piety, and faith. Thus, while the PAS may still exert an important effect on how Muslim women are perceived, these findings suggest that culture plays an important and highly influential role in how the PAS ultimately influences perceptions of others. Moreover, we offer fresh support for Dion et al.’s (1990) sociocultural perspective with data from a collectivist culture (UAE) that has not previously been investigated. It may be, therefore, that the suggestion for a Cultural Attractiveness Stereotype (or what might be referred to as the CAS) is more appropriate than the PAS when examining the perception of personal qualities of others in collectivist cultures. As millions of women around the world wear the hijab in their daily lives, further research is now required to uncover what other aspects of female person perception might also be influenced by wearing this garment.
